# Associations Between Insurance, Race and Ethnicity, and COVID-19 Hospitalization Beyond Underlying Health Conditions: A Retrospective Cohort Study

**DOI:** 10.1016/j.focus.2023.100120

**Published:** 2023-06-12

**Authors:** Kate H. McConnell, Anjum Hajat, Coralynn Sack, Stephen J. Mooney, Christine M. Khosropour

**Affiliations:** 1 Department of Epidemiology, School of Public Health, University of Washington, Seattle, Washington; 2 Department of Medicine, University of Washington, Seattle, Washington; 3 Department of Environmental & Occupational Health Sciences, University of Washington, Seattle, Washington

**Keywords:** COVID-19, social determinants of health, comorbidity, young adult, electronic health records

## Abstract

**Introduction:**

People of lower socioeconomic position and people of color experience higher risks of severe COVID-19, but understanding of these associations beyond the effect of underlying health conditions is limited. Moreover, few studies have focused on young adults, who have had the highest incidence of COVID-19 during much of the pandemic.

**Methods:**

We conducted a retrospective cohort study using electronic health record data from the University of Washington Medicine healthcare system. Our study population included individuals aged 18–39 years who tested positive for SARS-CoV-2 from February 2020 to March 2021. Using regression modeling, we estimated adjusted risk ratios and adjusted risk differences of COVID-19 hospitalization by socioeconomic position (measured by health insurance status) and race and ethnicity. We adjusted for any underlying health condition to examine these associations beyond the effect of underlying health conditions.

**Results:**

Among 3,101 individuals, the uninsured/publicly insured had a 1.9-fold higher risk of hospitalization (adjusted risk ratio=1.9, 95% CI=1.0, 3.6) and 9 additional hospitalizations per 1,000 SARS-CoV-2–positive persons (adjusted risk difference=9, 95% CI= −1, 20) than the privately insured. Hispanic or Latine, non-Hispanic Asian, non-Hispanic Black, and non-Hispanic Native Hawaiian or Pacific Islander patients had a 1.5-, 2.7-, 1.4-, and 2.1-fold higher risk of hospitalization (adjusted risk ratio=1.5, 95% CI=0.7, 3.1; 2.7, 95% CI=1.1, 6.5; 1.4, 95% CI=0.6, 3.3; and 2.1, 95% CI=0.5, 9.1), respectively, than non-Hispanic White patients.

**Conclusions:**

Although they should be interpreted cautiously given low precision, our findings suggest that the increased risk of COVID-19 hospitalization among young adults of lower socioeconomic position and young adults of color may be driven by forces other than underlying health conditions, including social determinants of health.

## Highlights

• In healthy young adults, severe COVID-19 is associated with public/no insurance.
• Severe COVID-19 risk is higher in young adults of color regardless of health status.
• Social determinants of health likely drive elevated risk of severe COVID-19.

## INTRODUCTION

Most people with coronavirus disease 2019 (COVID-19)—caused by severe acute respiratory syndrome coronavirus 2 (SARS-CoV-2)—experience mild or moderate illness, but some develop severe outcomes requiring hospitalization.^1^ In the U.S., the disproportionate impact of severe COVID-19 on people of color (POC) is well documented.^2^ POC have experienced higher rates of COVID-19–associated emergency department visits and hospitalizations and a disproportionate burden of COVID-19 deaths compared with non-Hispanic (NH) White individuals.^3^, ^4^, ^5^, ^6^, ^7^, ^8^, ^9^ In addition, COVID-19–associated hospitalizations and mortality have disproportionately impacted people of lower individual-level socioeconomic position (SEP), including people with lower incomes, people without a high school education, uninsured or publicly insured individuals, and people experiencing homelessness.^10^, ^11^, ^12^, ^13^

Frequently, these disparities in severe COVID-19 outcomes are attributed to higher rates of underlying health conditions (UHCs) that increase the risk of severe COVID-19^14^ among POC^15^, ^16^, ^17^, ^18^ and people of lower SEP.^18^^,^^19^ However, an emphasis on UHCs may underplay the importance of social determinants of health (SDOH).^20^ SDOH—such as experienced racism; limited healthcare access and utilization; disproportionate representation in essential work settings; living in higher-density and multigenerational households; higher rates of unemployment; and inequities in education, income, and wealth—prevent POC and people of lower SEP from having fair opportunities for health, including in the context of the COVID-19 pandemic.^21^, ^22^, ^23^, ^24^ Research exploring differences in severe COVID-19 outcomes between racial, ethnic, and socioeconomic groups beyond the effect of UHCs is needed to better understand the role of SDOH.

As with POC and people of lower SEP, young adults are more likely to experience lack of health insurance and limited healthcare access and utilization.^25^ In the U.S., adults aged <40 years have represented a substantial proportion of COVID-19 hospitalizations^26^ and have had the highest incidence of COVID-19 cases during much of the pandemic.^27^ However, there is limited research examining the associations between race and ethnicity, SEP, and COVID-19 severity in young adult populations, which would inform targeted COVID-19 prevention activities.

Mirroring national trends, in Washington (WA) State, POC have experienced higher rates of COVID-19–associated hospitalizations and deaths than NH White individuals,^28^ and adults aged <40 years have made up a notable percentage of COVID-19 hospitalizations^29^ and have had the highest incidence of COVID-19 cases throughout much of the pandemic.^30^ The aim of our study was to examine the associations between race and ethnicity, SEP, and COVID-19–associated hospitalization, above and beyond the effect of any diagnosed UHC, among young adult patients in a large academic medical system in western WA State.

## METHODS

We conducted a retrospective cohort study using electronic health record (EHR) data from the University of Washington Medicine (UWM) healthcare system, which is the largest academic medical system in the Central Puget Sound region. The Central Puget Sound region is located in western WA State and includes the Seattle metropolitan area; it comprises 56% of the state's population^31^ and, as of November 2022, about 54% of WA State's confirmed and probable COVID-19 cases.^30^ The University of Washington IRB approved this study and waived the informed consent and HIPAA authorization requirements (IRB identification: STUDY00011233). This study was unfunded.

### Study Population

Our study population included living patients aged 18–39 years engaged in care in the UWM system who tested positive for SARS-CoV-2 within UWM. SARS-CoV-2 testing began within UWM on February 29, 2020. Using EHR data, we identified patients who had at least 1 encounter in the UWM system from January 1, 2017 to February 28, 2020; who had at least 1 positive SARS-CoV-2 reverse transcription polymerase chain reaction (RT-PCR) test within UWM from February 29, 2020 to March 13, 2021 (14 days before the date of the data pull, to allow time for the occurrence of COVID-19–associated hospitalization after a positive test); and who were alive and aged 18–39 years at the time of their first positive SARS-CoV-2 RT-PCR test. This cohort of eligible patients included 3,251 individuals.

### Measures

We collected data at the patient level on health insurance status, race and ethnicity, any UHC, COVID-19–associated hospitalization, age (in years) at the time of first positive SARS-CoV-2 RT-PCR test, and sex assigned at birth (female/male). Of 3,251 eligible patients, we excluded 148 (4.6%) who were missing health insurance data and 2 (<0.1%) who had sex assigned at birth recorded as unknown, for a final analytic population of 3,101 individuals.

Studies have demonstrated the validity of EHR data for identifying health insurance status,^32^ which we used as a measure of individual-level SEP (uninsured or public versus private). A priori*,* we intended to analyze uninsured and public as separate categories but ultimately combined them because of the small proportion of uninsured patients in our analytic population (5.3%, *n*=165); other studies have taken a similar approach.^33^ The uninsured or public category included patients without health insurance and patients with Medicaid, Medicare (Part A and/or B only), public military insurance, and other forms of public insurance. The private category included patients with Medicare Advantage (Part C), Medicare Supplement (Medigap), private military insurance, employer-sponsored and individual plans, and other forms of private insurance.^34^^,^^35^ We based health insurance status on the most recent encounter between January 1, 2017 and February 28, 2020 for which insurance data were available.

Ethnicity data in the EHR system were originally recorded as either Hispanic or Latino or not Hispanic or Latino, and race data were originally recorded as American Indian or Alaska Native, Asian, Black or African American, Native Hawaiian or Other Pacific Islander, or White. Consistent with the racial and ethnic categories used by the WA State Department of Health when reporting COVID-19 data,^30^ we categorized race and ethnicity in our study as Hispanic or Latine, NH American Indian or Alaska Native (AIAN), NH Asian, NH Black, NH Native Hawaiian or Pacific Islander (NHPI), and NH White. We categorized race and ethnicity data that were missing or recorded as declined to answer, unavailable, or unknown as not recorded.

We defined any UHC (yes/no) as documented diagnosis of at least 1 UHC identified by the U.S. Centers for Disease Control and Prevention as conclusively associated with an increased risk of severe COVID-19 (Appendix Table 1, available online).^14^ To identify documented UHCs, we evaluated EHR data from January 1, 2017 to the date of the first positive SARS-CoV-2 RT-PCR test. We identified most UHCs exclusively by relevant ICD-10-CM diagnosis codes. We categorized patients who had no indication of any UHCs as having no UHCs.

We defined COVID-19–associated hospitalization (yes/no) as an inpatient encounter within UWM that either (1) had an admit date at most 3 days before the date of a positive SARS-CoV-2 test and an indication in the admit note that the patient was hospitalized for COVID-19 or (2) was within 14 days after a positive SARS-CoV-2 test; this is the standard approach validated and used by the University of Washington Institute of Translational Health Sciences to identify COVID-19–associated hospitalizations.

### Statistical Analysis

We calculated the unadjusted proportions of patients who experienced COVID-19–associated hospitalization by health insurance status and race and ethnicity, overall and stratified by any UHC. We estimated 95% CIs using the Wilson score interval with Yates’ continuity correction. We present these data for all racial and ethnic groups but do not consider them sufficient to draw conclusions about the risk of COVID-19–associated hospitalization for (1) NH AIAN patients or (2) NH Asian and NH NHPI patients without UHCs because of small sample sizes and/or 0 COVID-19–associated hospitalizations in these groups. In addition, we present but do not interpret these data for patients with unrecorded race and ethnicity because of the likely heterogeneity of this group.

In addition, we estimated adjusted risk ratios (aRRs) and adjusted risk differences (aRDs) of COVID-19–associated hospitalization by health insurance status and race and ethnicity, adjusted for any UHC to examine these associations above and beyond the effect of any diagnosed UHC. We also adjusted for the following potential confounders: sex assigned at birth, continuous age, and race and ethnicity in the health insurance models; and sex assigned at birth and continuous age in the race and ethnicity models. We did not adjust for health insurance status in the race and ethnicity models because differences in SEP across racial and ethnic groups are an important contributor to racial and ethnic disparities in health.^36^^,^^37^ We used patients with private insurance and NH White patients as the referent groups because we expected these groups to have the lowest risks. In the race and ethnicity regression models, we did not include 29 patients (0.9%) recorded as NH AIAN because insufficient data (small numbers and 0 outcomes) would have resulted in an unreliable parameter estimate for this group, and we did not include 447 patients (14.4%) with unrecorded race and ethnicity because this parameter estimate would have been uninterpretable given the probable heterogeneity of this group. We estimated aRRs using multivariable log-binomial regression and aRDs using generalized linear regression with a Gaussian distribution and identity link function, both with Huber–White estimates of the SE.^38^

## RESULTS

Among 3,101 SARS-CoV-2–positive patients aged 18–39 years, the average age at the time of first positive SARS-CoV-2 RT-PCR test was 27.9 years (SD=6.2 years); 18.0% of patients were recorded as Hispanic or Latine, and 13.5% were recorded as NH Black; 52.8% were assigned female sex at birth; and 42.8% had at least 1 documented UHC (Table 1). Overall, 46 patients (1.5%) experienced COVID-19–associated hospitalization (Appendix Table 2, available online).

**Table 1:** Characteristics of SARS-CoV-2–Positive Patients Aged 18–39 Years, by Insurance Status, University of Washington Medicine, February 2020–March 2021

	Total^a^ N=3,101		Uninsured or public health insurance *n*=1,168		Private health insurance *n*=1,933	
Variable	*n*	%	*n*	%	*n*	%
Age (years)^b^ (mean, SD)	27.9	6.2	29.1	6.1	27.1	6.2
Race and ethnicity						
Hispanic or Latine	557	18.0	323	27.7	234	12.1
NH AIAN	29	0.9	16	1.4	13	0.7
NH Asian	277	8.9	66	5.7	211	10.9
NH Black	418	13.5	265	22.7	153	7.9
NH NHPI	56	1.8	26	2.2	30	1.6
NH White	1,317	42.5	332	28.4	985	51.0
Not recorded	447	14.4	140	12.0	307	15.9
Sex^c^						
Female	1,636	52.8	688	58.9	948	49.0
Male	1,465	47.2	480	41.1	985	51.0
Any UHC^d^						
No	1,773	57.2	538	46.1	1,235	63.9
Yes	1,328	42.8	630	53.9	698	36.1

a A total of 148 patients (4.6%) were missing health insurance data and excluded from the final analytic data set.

b Age at first positive SARS-CoV-2 test within the University of Washington Medicine healthcare system.

c Sex assigned at birth; 2 patients (<0.1%) had sex assigned at birth recorded as unknown and were excluded from the final analytic data set.

d See Appendix Table 1 (available online) for a list of UHCs. AIAN, American Indian or Alaska Native; NH, non-Hispanic; NHPI, Native Hawaiian or Pacific Islander; UHC, underlying health condition.

Patients who were uninsured or publicly insured (*n*=1,168, 37.7%) were slightly older and had a higher proportion of individuals with documented diagnosis of at least 1 UHC than privately insured patients (*n*=1,933, 62.3%) (Table 1). Patients recorded as Hispanic or Latine, NH AIAN, NH Black, or NH NHPI comprised larger proportions of the uninsured or publicly insured group than the privately insured group. Table 2 presents patient characteristics by race and ethnicity. Patients recorded as Hispanic or Latine, NH AIAN, NH NHPI, and NH Black had higher proportions of individuals who were uninsured or publicly insured and higher proportions of individuals with at least 1 UHC than NH Asian and NH White patients.

**Table 2:** Characteristics of SARS-CoV-2–Positive Patients Aged 18–39 Years, by Race and Ethnicity, University of Washington Medicine, February 2020–March 2021

	Total N=3,101		Hispanic or Latine *n*=557		NH AIAN *n*=29		NH Asian *n*=277		NH Black *n*=418		NH NHPI *n*=56		NH White *n*=1,317		Not recorded *n*=447	
Variable	*n*	%	*n*	%	*n*	%	*n*	%	*n*	%	*n*	%	*n*	%	*n*	%
Age (years)^a^ (mean, SD)	27.9	6.2	29.2	5.9	29.5	6.2	27.2	6.0	28.6	6.2	28.8	6.6	27.0	6.3	28.4	6.1
Insurance^b^																
Private	1,933	62.3	234	42.0	13	44.8	211	76.2	153	36.6	30	53.6	985	74.8	307	68.7
Uninsured or public	1,168	37.7	323	58.0	16	55.2	66	23.8	265	63.4	26	46.4	332	25.2	140	31.3
Sex^c^																
Female	1,636	52.8	325	58.3	16	55.2	143	51.6	218	52.2	25	44.6	682	51.8	227	50.8
Male	1,465	47.2	232	41.7	13	44.8	134	48.4	200	47.8	31	55.4	635	48.2	220	49.2
Any UHC^d^																
No	1,773	57.2	262	47.0	11	37.9	180	65.0	227	54.3	20	35.7	791	60.1	282	63.1
Yes	1,328	42.8	295	53.0	18	62.1	97	35.0	191	45.7	36	64.3	526	39.9	165	36.9

a Age at first positive SARS-CoV-2 test within the University of Washington Medicine healthcare system.

b A total of 48 patients (4.6%) were missing health insurance data and excluded from the final analytic data set.

c Sex assigned at birth; 2 patients (<0.1%) had sex assigned at birth recorded as unknown and were excluded from the final analytic data set.

d See Appendix Table 1 (available online) for a list of UHCs. AIAN, American Indian or Alaska Native; NH, non-Hispanic; NHPI, Native Hawaiian or Pacific Islander; UHC, underlying health condition.

**Figure 1:**
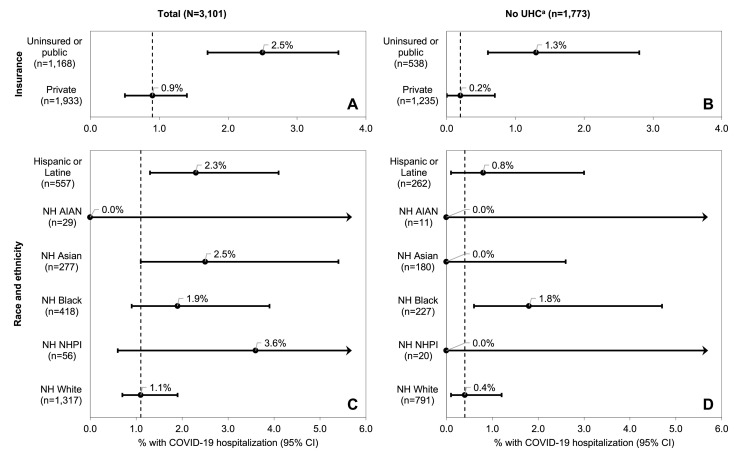
Unadjusted proportions of patients who experienced COVID-19 hospitalization, by insurance status and race and ethnicity.^b^ *Note:* Proportions by health insurance status are provided for the entire study population in Panel A and in the stratum without any UHC in Panel B, proportions by race and ethnicity are provided for the entire study population in Panel C and in the stratum without any UHC in Panel D, and proportions in the stratum with any UHC and among patients with unrecorded race and ethnicity are given in Appendix Table 2 (available online). The 95% CIs were calculated using the Wilson score interval with Yates’ continuity correction. The vertical dashed lines represent the risk of COVID-19–associated hospitalization in patients with private health insurance in the insurance plots and the risk in NH White patients in the race and ethnicity plots. ^a^See Appendix Table 1 (available online) for a list of UHCs. ^b^Among SARS-CoV-2–positive patients aged 18–39 years within the University of Washington Medicine healthcare system, February 2020–March 2021. AIAN, American Indian or Alaska Native; NH, non-Hispanic; NHPI, Native Hawaiian or Pacific Islander; UHC, underlying health condition.

Overall, the risk of COVID-19–associated hospitalization was higher among patients who were uninsured or publicly insured than among those who were privately insured (2.5%, 95% CI=1.7%, 3.6% vs 0.9%, 95% CI=0.5%, 1.4%) (Figure 1 and Appendix Table 2, available online). Similarly, among patients without UHCs, the proportion of patients who developed COVID-19–associated hospitalization was higher among the uninsured or publicly insured (1.3%) than among the privately insured (0.2%), although the estimated 95% CIs overlapped. After adjustment for potential confounders and any UHC, patients who were uninsured or publicly insured had a 1.9-fold higher risk of COVID-19–associated hospitalization (aRR=1.9, 95% CI=1.0, 3.6) and 9 additional hospitalizations per 1,000 SARS-CoV-2–positive persons during the 13-month study period (aRD=9, 95% CI= −1, 20) than patients on private health insurance, although the 95% CIs were wide and contained the null values (Table 3).

**Table 3:** Risks, Risk Ratios, and Risk Differences of COVID-19 Hospitalization by Insurance Status and Race and Ethnicity

Variable^a^	Risk of COVID-19 hospitalization		Crude RR		Adjusted RR^b^		Crude RD^c^		Adjusted RD^b^^,^^c^	
	*n*/N	%	RR	95% CI	aRR	95% CI	RD	95% CI	aRD	95% CI
Insurance										
Uninsured or public	29/1,168	2.5	2.8	1.6, 5.1	1.9	1.0, 3.6	16	6, 26	9	−1, 20
Private	17/1,933	0.9	ref		ref		ref		ref	
Race and ethnicity										
Hispanic or Latine	13/557	2.3	2.0	1.0, 4.3	1.5	0.7, 3.1	12	−2, 26	5	−9, 19
NH Asian	7/277	2.5	2.2	0.9, 5.4	2.7	1.1, 6.5	14	−5, 33	15	−5, 34
NH Black	8/418	1.9	1.7	0.7, 3.9	1.4	0.6, 3.3	8	−7, 22	4	−10, 19
NH NHPI	2/56	3.6	3.1	0.7, 13.4	2.1	0.5, 9.1	24	−25, 73	17	−31, 66
NH White	15/1,317	1.1	ref		ref		ref		ref	

*Note:* Data shown are among SARS-CoV-2–positive patients aged 18–39 years within the University of Washington Medicine healthcare system, February 2020–March 2021. RRs were estimated by log-binomial regression using Huber–White estimates of the SE; RDs were estimated using a generalized linear model with a Gaussian distribution and identity link function and using Huber–White estimates of the SE.

a A total of 29 patients (0.9%) recorded as NH AIAN were not included in the race and ethnicity regression models because insufficient data (small numbers and 0 outcomes) would have resulted in an unreliable parameter estimate for this group; 447 patients (14.4%) with unrecorded race and ethnicity were also not included in the race and ethnicity regression models because this parameter estimate would have been uninterpretable given the probable heterogeneity of this group.

b Insurance aRR and aRD models adjusted for documented diagnosis of any UHC (yes/no) (see Appendix Table 1, available online, for a list of UHCs), sex assigned at birth (female/male), continuous age (years), and race and ethnicity (Hispanic or Latine, NH AIAN, NH Asian, NH Black, NH NHPI, NH White, not recorded). Race and ethnicity aRR and aRD models adjusted for any UHC, sex, and age.

c Difference in cumulative incidence per 1,000 SARS-CoV-2–positive persons over the 13-month study period. AIAN, American Indian or Alaska Native; aRD, adjusted risk difference; aRR, adjusted risk ratio; NH, non-Hispanic; NHPI, Native Hawaiian or Pacific Islander; RD, risk difference; RR, risk ratio; UHC, underlying health condition.

Overall, 2.3% of patients recorded as Hispanic or Latine, 2.5% of NH Asian patients, 1.9% of NH Black patients, and 3.6% of NH NHPI patients experienced COVID-19–associated hospitalization compared with 1.1% of NH White patients (Figure 1 and Appendix Table 2, available online). Among patients without UHCs, higher proportions of Hispanic or Latine (0.8%) and NH Black patients (1.8%) experienced COVID-19–associated hospitalization compared with NH White patients (0.4%), although the estimated 95% CIs were generally wide and overlapping. After adjustment for potential confounders and any UHC, patients who were recorded as Hispanic or Latine, NH Asian, NH Black, or NH NHPI had a 1.5-, 2.7-, 1.4-, and 2.1-fold higher risk of COVID-19–associated hospitalization (aRR=1.5, 95% CI=0.7, 3.1; 2.7, 95% CI=1.1, 6.5; 1.4, 95% CI=0.6, 3.3; and 2.1, 95% CI=0.5, 9.1) and 5, 15, 4, and 17 additional hospitalizations per 1,000 SARS-CoV-2–positive persons during the 13-month study period (aRD=5, 95% CI= −9, 19; 15, 95% CI= −5, 34; 4, 95% CI= −10, 19; and 17, 95% CI= −31, 66), respectively, compared with patients recorded as NH White, although the 95% CIs were wide and most contained the null values (Table 3).

## DISCUSSION

We found that among SARS-CoV-2–positive adults aged <40 years without UHCs, a higher proportion of uninsured or publicly insured patients experienced COVID-19–associated hospitalization compared with privately insured patients, and higher proportions of patients recorded as Hispanic or Latine or NH Black experienced COVID-19–associated hospitalization compared with NH White patients. Similarly, although the 95% CIs were wide and most spanned the null values, the magnitudes and directions of our adjusted estimated measures of excess risk suggest that uninsured or publicly insured young adults and young adult POC may be at higher relative and absolute risk of COVID-19–associated hospitalization than privately insured and NH White young adults, above and beyond the effect of any UHC.

Previous studies have shown that POC and people of lower SEP are at higher risk of severe COVID-19 than NH White individuals^3^, ^4^, ^5^, ^6^, ^7^, ^8^, ^9^ and people of higher SEP.^10^, ^11^, ^12^, ^13^ Commentators have largely attributed this excess disease burden to POC and people of lower SEP (1) being more likely to be exposed to SARS-CoV-2 in part because of disproportionate representation in essential work settings and living in higher-density and multigenerational households^21^, ^22^, ^23^, ^24^ and (2) being at increased risk of severe COVID-19 because of higher rates of UHCs.^15^, ^16^, ^17^, ^18^, ^19^ Our study builds on this previous work by only including patients who tested positive for SARS-CoV-2 and either stratifying by or statistically adjusting for any UHC. Although they should be interpreted cautiously given low precision, our findings suggest that the differences in risk of COVID-19–associated hospitalization between young adults of lower SEP and those of higher SEP and between young adult POC and NH White young adults may be driven by forces other than UHCs.

Our analyses add to the limited body of research on racial and ethnic disparities in COVID-19 outcomes beyond the effect of UHCs, which shows that these disparities persist even after controlling for UHCs.^39^, ^40^, ^41^, ^42^, ^43^ Similar to our results, an analysis of EHR data from 21 states found racial and ethnic disparities in hospitalizations among COVID-19 cases even after controlling for certain sociodemographic factors and select UHCs, with NH Asian patients exhibiting the highest risk relative to NH White patients, followed by Hispanic patients, and NH Black patients.^39^ One possible explanation for these findings is that because of limited healthcare access and utilization,^22^ POC and people of lower SEP have higher rates of undiagnosed health conditions,^44^, ^45^, ^46^, ^47^, ^48^ creating the potential for disproportionate rates of severe COVID-19 even among those without diagnosed UHCs. Nevertheless, it is critical to also consider the role of SDOH in COVID-19 disparities, including residing in areas of higher deprivation; lower quality of clinical care; racism in healthcare; higher rates of unemployment; chronic stress from racial discrimination; and inequities in housing, transportation, education, income, and wealth, which previous studies have elucidated.^2^^,^^5^^,^^49^, ^50^, ^51^, ^52^

A novel element of our study is its focus on young adults, who have had the highest incidence of COVID-19 cases throughout much of the pandemic.^27^ Our findings highlight the importance of ensuring equitable access to COVID-19 resources, including vaccines and anti–SARS-CoV-2 therapy, for young adult POC and young adults of lower SEP in general, not only for those with UHCs.^53^, ^54^, ^55^ Furthermore, improved understanding of the demographic and clinical features of young adults with COVID-19 and of racial, ethnic, and SEP disparities in COVID-19 outcomes among young adults allows for targeted local morbidity and mortality prevention initiatives tailored to young adult populations, including culturally and linguistically appropriate risk communication and collaboration with community partners.

### Limitations

In addition to the innovative elements highlighted earlier, strengths of our study include leveraging EHR data from a large academic medical system and estimating both relative and absolute measures of excess risk.^56^ Our study also has several important limitations. A primary limitation is low precision, driven by insufficient sample size in the context of relatively low incidence of COVID-19–associated hospitalization among young adults. Even with several thousand patients included in our study, we only identified 56 recorded as NH NHPI and 29 recorded as NH AIAN—the 2 groups with the highest age-adjusted COVID-19 hospitalization and death rates in WA State as of November 2022.^28^ Second, we observed high levels of missingness for race and ethnicity, increasing the likelihood of selection bias in our race and ethnicity regression models. Because EHR data are often missing not at random,^57^^,^^58^ we did not employ multiple imputation methods, which may further increase bias under missing not at random conditions.^59^ Instead, we categorized missing race and ethnicity data as not recorded, which may have led to residual confounding and bias in our health insurance regression models.^60^^,^^61^ Furthermore, race and ethnicity data recorded in EHR systems may not be valid, especially for POC,^62^^,^^63^ leading to differential exposure misclassification. Third, health insurance may be an incomplete measure of individual-level SEP, leading to nondifferential exposure misclassification. Fourth, because POC and people of lower SEP have higher rates of undiagnosed health conditions,^44^, ^45^, ^46^, ^47^, ^48^ we may have disproportionately failed to identify UHCs among POC and uninsured or publicly insured patients. Fifth, we only captured COVID-19 hospitalizations within the UWM system, which may have led to differential outcome misclassification if the likelihood of COVID-19 hospitalization at an outside facility differed by racial and ethnic or health insurance group. Sixth, our analyses did not account for secular trends, such as changes in vaccine availability, testing practices, and circulating viral strains over time. Finally, we used data from a single academic medical center, which may limit the generalizability of our results. However, the proportion of patients in our study with any UHC was similar to the 2019 national estimate of the prevalence of chronic conditions in adults aged 18–34 years,^64^ which supports the generalizability of our findings.

## CONCLUSIONS

In conclusion, although they should be interpreted cautiously given low precision, our findings suggest the increased risk of COVID-19–associated hospitalization among SARS-CoV-2–positive young adults of lower SEP and young adult POC may be driven by forces other than an increased risk of UHCs, including SDOH. Future research should directly explore the relationships between SDOH and COVID-19 as well as other health outcomes in young adult populations to better understand upstream influences.^65^ COVID-19 has exacerbated existing health disparities across the U.S. Recognizing the roots of these disparities is essential to the development of targeted and effective community- and healthcare system–based public policies and health interventions that emphasize health equity and social justice.^65^

## CRediT authorship contribution statement

**Kate H. McConnell:** Conceptualization, Methodology, Software, Formal analysis, Investigation, Data curation, Writing – original draft, Visualization, Project administration. **Anjum Hajat:** Conceptualization, Writing – review & editing, Supervision. **Coralynn Sack:** Conceptualization, Writing – review & editing. **Stephen J. Mooney:** Conceptualization, Writing – review & editing. **Christine M. Khosropour:** Conceptualization, Methodology, Writing – review & editing, Supervision.

## Appendix Supplementary materials

Image, application 1

## Footnotes

Supplementary material associated with this article can be found in the online version at doi:10.1016/j.focus.2023.100120.
