# Supplementary material for: Associations Between Insurance, Race and Ethnicity, and COVID-19 Hospitalization Beyond Underlying Health Conditions: A Retrospective Cohort Study
Source: AJPM Focus. 2023 Jun 12;2(3):100120. doi: 10.1016/j.focus.2023.100120 (PMC10260262; doi:10.1016/j.focus.2023.100120)
Supplement: Supplementary file 1 [file mmc1.pdf]

# APPENDIX TABLES

**Appendix Table 1.** UHCs conclusively associated with increased risk of severe COVID-19 per the U.S. Centers for Disease Control and Prevention and identifying ICD-10-CM codes

| UHC[a] | ICD-10-CM code[b] | ICD-10-CM description |
|---|---|---|
| Bronchiectasis | J47 | Bronchiectasis |
| Cancer | C00-C96 | Malignant neoplasms |
| Cerebrovascular disease | I60-I69 | Cerebrovascular diseases |
| Chronic kidney disease | N18 | Chronic kidney disease (CKD) |
| Chronic obstructive pulmonary disorder | J44 | Other chronic obstructive pulmonary disease |
| Corticosteroids or other immunosuppressive medications | D84.821; Z79.52; Z92.25 | Immunodeficiency due to drugs; Long term (current) use of systemic steroids; personal history of immunosuppression therapy |
| Cystic fibrosis | E84 | Cystic fibrosis |
| Diabetes mellitus, type 1 | E10 | Type 1 diabetes mellitus |
| Diabetes mellitus, type 2 | E11 | Type 2 diabetes mellitus |
| Down syndrome | Q90 | Down syndrome |
| Heart conditions (e.g., heart failure, coronary artery disease, cardiomyopathies) | I20-I25; I30-I52 | Ischemic heart disease; Other forms of heart disease |
| Human immunodeficiency virus | B20; Z21 | Human immunodeficiency virus (HIV) disease; Asymptomatic human immunodeficiency virus (HIV) infection status |
| Immune deficiencies | D80; D81; D82; D83; D84.0; D84.1; D84.81; D84.822; D84.89; D84.9 | Immunodeficiency with predominantly antibody defects; Combined immunodeficiencies; Immunodeficiency associated with other major defects; Common variable immunodeficiency; Lymphocyte function antigen-1 [LFA-1] defect; Defects in the complement system; Immunodeficiency due to conditions classified elsewhere; Immunodeficiency due to external causes; Other immunodeficiencies; Immunodeficiency, unspecified |
| Liver disease, chronic (cirrhosis, non-alcoholic fatty liver disease, alcoholic liver disease, autoimmune hepatitis) | K70-K77 | Diseases of liver |
| Lung disease, interstitial | J80-J84 | Other respiratory diseases principally affecting the interstitium |
| Lung disease, other | I28; J41; J42; J43; J60-J70; J85-J86; J90-J94; Z99.81 | Other diseases of pulmonary vessels; Simple and mucopurulent chronic bronchitis; Unspecified chronic bronchitis; Emphysema; Lung diseases due to external agents; Suppurative and necrotic conditions of the lower respiratory tract; Other diseases of the pleura; Dependence on supplemental oxygen |
| Mental health disorders (mood disorders, e.g., depression and schizophrenia spectrum disorders) | F20-F29; F30-F39 | Schizophrenia, schizotypal, delusional, and other non-mood psychotic disorders; Mood (affective) disorders |
| Neurologic conditions | F01; F02; F03; G10-G14; G20; G30-G32; G35; G45; G70; G80-G83 | Vascular dementia; Dementia in other diseases classified elsewhere; Unspecified dementia; Systemic atrophies primarily affecting the central nervous system; Parkinson’s disease; Other degenerative diseases of the nervous system; Multiple sclerosis; Transient cerebral ischemic attacks and related syndromes; Myasthenia gravis and other myoneural disorders; Cerebral palsy and other paralytic syndromes |

**Appendix Table 1.** UHCs conclusively associated with increased risk of severe COVID-19 per the U.S. Centers for Disease Control and Prevention and identifying ICD-10-CM codes

| **UHC**[a] | **ICD-10-CM code**[b] | **ICD-10-CM description** |
|---|---|---|
| Obesity (BMI ≥30 $kg/m^2$)[c] | E66.0; E66.1; E66.2; E66.8; E66.9 | Obesity due to excessive calories; Drug-induced obesity; Morbid (severe) obesity with alveolar hypoventilation; Other obesity; Obesity, unspecified |
| Pregnancy and recent pregnancy | Z33; Z34; Z36; Z37; Z38; Z39; Z3A | Pregnant state; Encounter for supervision of normal pregnancy; Encounter for antenatal screening of mother; Outcome of delivery; Liveborn infants according to place of birth and type of delivery; Encounter for maternal postpartum care and examination; Weeks of gestation |
| Pulmonary hypertension and pulmonary embolism | I26; I27 | Pulmonary embolism; Other pulmonary heart disease |
| Smoking, current and former[c] | F17; Z72.0; Z87.891 | Nicotine dependence; Tobacco use; Personal history of nicotine dependence |
| Solid organ or blood stem cell transplantation | Z94; T86 | Transplanted organ and tissue status; Complications of transplanted organs and tissue |
| Tuberculosis | A15 | Respiratory tuberculosis |

[a]UHCs for which there is a published meta-analysis or systematic review demonstrating a conclusive increase in risk of severe COVID-19 according to the U.S. Centers for Disease Control and Prevention.[1]

[b]ICD-10-CM codes and other EHR data for all underlying health conditions were evaluated from 01/01/2017 to the date of the first positive SARS-CoV-2 RT-PCR test, except those for pregnancy and recent pregnancy, which were evaluated for the 60 days prior to the date of the first positive SARS-CoV-2 RT-PCR test.

[c]In addition to the ICD-10-CM codes indicated, EHR height and weight data were used to calculate BMI to identify patients with obesity, and EHR smoking data were used to identify patients with a history of smoking. BMI was based on the most recent encounter between 01/01/2017 and the date of the first positive SARS-CoV-2 RT-PCR test for which height, weight, and the calculated BMI were biologically plausible. Biological plausibility for height was defined as ≥1.2m and ≤2.4m, for weight as ≥34.0kg and ≤272.2kg, and for BMI as ≤80.0$kg/m^2$.[2] Patients who had a smoking status of current or former smoker recorded at any encounter between 01/01/2017 and the date of the first positive SARS-CoV-2 RT-PCR test were classified as current or former smokers in our analyses.

BMI, body mass index; EHR, electronic health record; RT-PCR, reverse transcription polymerase chain reaction; UHC, underlying health condition.

**Appendix Table 2.** Unadjusted proportions of SARS-CoV-2-positive patients aged 18-39 years who experienced COVID-19 hospitalization, by insurance status and race and ethnicity, overall and stratified by any UHC, University of Washington Medicine healthcare system, February 2020-March 2021

| **Variable** | | **Risk of COVID-19 hospitalization** | | | | | | | | |
|---|---|---|---|---|---|---|---|---|---|---|
| | | **Total (N = 3,101)** | | | **No UHC[a] (*n* = 1,773)** | | | **Any UHC[a] (*n* = 1,328)** | | |
| | | ***n*/N** | **%** | **95% CI** | ***n*/N** | **%** | **95% CI** | ***n*/N** | **%** | **95% CI** |
| Insurance | Uninsured or public | 29 / 1,168 | 2.5 | 1.7, 3.6 | 7 / 538 | 1.3 | 0.6, 2.8 | 22 / 630 | 3.5 | 2.3, 5.3 |
| | Private | 17 / 1,933 | 0.9 | 0.5, 1.4 | 2 / 1,235 | 0.2 | 0.0, 0.7 | 15 / 698 | 2.1 | 1.3, 3.6 |
| Race and ethnicity | Hispanic or Latine | 13 / 557 | 2.3 | 1.3, 4.1 | 2 / 262 | 0.8 | 0.1, 3.0 | 11 / 295 | 3.7 | 2.0, 6.8 |
| | NH AIAN | 0 / 29 | 0.0 | 0.0, 14.6 | 0 / 11 | 0.0 | 0.0, 32.1 | 0 / 18 | 0.0 | 0.0, 21.9 |
| | NH Asian | 7 / 277 | 2.5 | 1.1, 5.4 | 0 / 180 | 0.0 | 0.0, 2.6 | 7 / 97 | 7.2 | 3.2, 14.8 |
| | NH Black | 8 / 418 | 1.9 | 0.9, 3.9 | 4 / 227 | 1.8 | 0.6, 4.7 | 4 / 191 | 2.1 | 0.7, 5.6 |
| | NH NHPI | 2 / 56 | 3.6 | 0.6, 13.4 | 0 / 20 | 0.0 | 0.0, 20.0 | 2 / 36 | 5.6 | 1.0, 20.0 |
| | Not recorded | 1 / 447 | 0.2 | 0.0, 1.4 | 0 / 282 | 0.0 | 0.0, 1.7 | 1 / 165 | 0.6 | 0.0, 3.8 |
| | NH White | 15 / 1,317 | 1.1 | 0.7, 1.9 | 3 / 791 | 0.4 | 0.1, 1.2 | 12 / 526 | 2.3 | 1.2, 4.1 |

The 95% CIs were calculated using the Wilson score interval with Yates' continuity correction.
[a]See Appendix Table 1 for a list of UHCs.
AIAN, American Indian or Alaska Native; NH, non-Hispanic; NHPI, Native Hawaiian or Pacific Islander; UHC, underlying health condition.

## REFERENCES


1. COVID-19 - Underlying Medical Conditions. Centers for Disease Control and Prevention. https://www.cdc.gov/coronavirus/2019-ncov/science/science-briefs/underlying-evidence-table.html. Published 2022. Accessed March 16, 2022.

2. Littman AJ, Boyko EJ, McDonell MB, Fihn SD. Evaluation of a Weight Management Program for Veterans. *Prev Chronic Dis*. 2012;9:110267. doi:http://dx.doi.org/10.5888/pcd9.110267
